# Emergency and routine presentation of neuroendocrine neoplasia in England: determinants of late presentation and survival outcomes

**DOI:** 10.1530/EO-25-0012

**Published:** 2025-04-14

**Authors:** Marie Line El Asmar, Mohamed Mortagy, Benjamin E White, Dan Burns, John Ramage

**Affiliations:** ^1^ Department of Gastroenterology, Hampshire Hospitals NHS Foundation Trust, Basingstoke, UK; ^2^ Hampshire Hospitals NHS Foundation Trust, Winchester, UK; ^3^ St. George University School of Medicine, University Centre Grenada, West Indies, Grenada; ^4^ Kings Health Partners NET Centre, Kings College Hospital, London, UK; ^5^ School of Electronics and Computer Science, University of Southampton, Southampton, UK; ^6^ NIHR Applied Research Collaboration Wessex, University of Southampton, Chilworth, Southampton, UK; ^7^ University of Winchester, Basingstoke, Hampshire, UK

**Keywords:** neuroendocrine neoplasia, presentation, diagnosis, neuroendocrine tumour, neuroendocrine tumour, neuroendocrine carcinoma, NET, NEC, survival, machine learning, XGBoost

## Abstract

**Objective:**

The time from onset of symptoms of neuroendocrine neoplasia (NEN) to diagnosis ranges between 5 and 7 years. Risk factors associated with this and the difference in overall survival (OS) between routine and emergency presentation (RP and EP) are not known.

**Design:**

A retrospective, population-based study.

**Methods:**

A retrospective, population-based study of gastroenteropancreatic and lung NEN registered on England’s national cancer between 2012 and 2021. Factors associated with worse OS, or emergency or late presentation (EP or LP), were evaluated using the Kaplan–Meier estimator, Cox and logistic regressions, and machine learning (ML) in two models.

**Results:**

A total of 21,345 NEN were included. 20.3% were EP. EP showed worse OS compared to RP. Factors associated with EP were male sex, advanced stage, worse deprivation and NEC. The ML model showed EP related to advanced stage, small intestinal NEN, NEC, advanced age, deprivation and male sex in decreasing order of importance. Factors associated with LP included EP, male sex and NEC. The ML model showed that NEC, small intestinal NEN, advanced age, EP and male sex are associated with LP in decreasing order of importance.

**Conclusion:**

EP is associated with poor survival. Addressing the associated factors may aid in timely diagnosis and improved survival.

## Introduction

Neuroendocrine neoplasms (NENs) are a group of rare cancers predominantly found in the bronchopulmonary and gastrointestinal systems. NEN incidence has been increasing globally (White *et al.* 2022). NEN can be categorised into well-differentiated neuroendocrine tumours (NETs) and poorly differentiated, high-grade neuroendocrine carcinomas (NECs) (Rindi *et al.* 2018).

Gastroenteropancreatic NEN (GEPNEN) characteristically present between the ages 50 and 60 (Horton *et al.* 2004), and can be difficult to diagnose, as they either present asymptomatically and are diagnosed incidentally (e.g. post-appendicectomy), as non-specific bowel symptoms causing delays in diagnosis or with symptoms resulting from mass effect of the tumour within the abdomen (Oronsky *et al.* 2017, Tadman *et al.* 2019). About 10% present with a classical carcinoid syndrome (Modlin *et al.* 2005). Research suggests that the time from the onset of symptoms to the diagnosis of NEN averages 5–7 years (Modlin *et al.* 2005). This process often involves several visits to the general practitioner (GP) and frequent misdiagnoses such as irritable bowel syndrome or gastritis. Lung NEN may present with cough, wheeze, shortness of breath, chest pain or haemoptysis, depending on tumour aggressiveness and location, or may be found incidentally on imaging (Ramage *et al.* 2005, Tadman *et al.* 2019).

Route to diagnosis is recorded for patients with NEN within England’s National Cancer Registry and Analysis Service (NCRAS) database. The emergency pathway for the diagnosis and initial treatment of NEN can be regarded as a proxy for a delayed diagnosis, as it suggests that the case has not undergone diagnostic evaluation at an earlier phase of symptom presentation. Disease stage at presentation may also reflect delays in diagnosis. The factors in NEN associated with emergency presentations or late presentation are unclear. In one study, 80% reportedly had GP encounters with symptoms before diagnosis over a mean duration of 37 months and a mean of 11 interactions per patient (Basuroy *et al.* 2018). Knowledge of factors leading to NEN presentation helps health commissioners target populations at higher risk to reduce delays to diagnosis. Addressing delays and inequity in the timely diagnosis of NEN aligns with targets set by the NHS Long Term Plan and the UK Rare Diseases Framework (Department of Health and Social Care 2021).

This article aims to assess factors associated with emergency versus routine presentation at the time of diagnosis of NEN, to assess factors associated with late presentation (higher stage) at diagnosis of NEN and to correlate these with overall survival.

## Methods

### Data extraction

A population-based study using retrospective data from the National Cancer Registration and Analysis Service (NCRAS). NCRAS collects data on all diagnosed tumours within the NHS in England (National Cancer Registration and Analysis Service 2025). A total of 21,345 cases of GEP-NEN (excluding appendix NENs) and lung NEN occurring between 2012 and 2021 were included in the analysis. NEN at the following anatomical sites C16.0-C16.9, C17.0-C17.9, C18.0, C18.2-C18.9, C20.9, C25.0-C25.9 and C34.0-C34.9 were extracted using the International Statistical Classification of Diseases and Related Health Problems, 10th Revision (ICD-10) (World Health Organization 2016). Morphology codes included 8013, 8041–8045 (excluding lung), 8150–8158 (excluding 8154), 8240–8246 (excluding 8244), 8249 and 9091 according to the International Classification of Diseases for Oncology, 3rd Edition (ICD-O-3) (World Health Organization 2019). Appendix NENs were excluded as they present incidentally mimicking appendicitis, diagnosed incidentally at histopathology as stage 1 resected tumour, which could potentially skew results. Cases without survival follow-up time or with zero survival time, or without routes to diagnosis data, and those recorded as ‘death certificate only’ were excluded. Routes to diagnosis data were grouped as emergency or routine presentation. Routine presentation included GP referrals, elective inpatient admissions, other outpatient appointments, screening and 2-week wait referrals. Emergency presentations were admissions to the hospital via the emergency department or acute admissions units, which led directly to the diagnosis of NEN. Patients were classified into two groups as early (stage 1 and 2 at diagnosis) and late presentations (stage 3 and 4 at diagnosis).

All tumours were grouped by morphology into well-differentiated neuroendocrine tumours (NETs) or poorly differentiated neuroendocrine carcinomas (NECs). The pre-diagnosis variables included in the analysis were sex, age, index of multiple deprivation (IMD), ethnicity, rurality and region. Post-diagnosis variables included morphology, stage and site. Tumour size and grade were excluded from the analysis due to a significant number of missing data. IMD is a composite of seven domains producing a surrogate measure of deprivation, comprising income (22.5%), employment (22.5%), educational, skills and training (13.5%), health (13.5%), crime (9.3%), housing (9.3%) and environment (9.3%) (Ministry of Housing, Communities and Local Government 2019).

### Statistical analysis

#### Descriptive analysis

Descriptive data included categorical variables presented in frequencies and percentages. A Pearson’s chi-squared test evaluated the differences between the emergency and routine presentation groups. A *P*-value <0.05 was deemed statistically significant. All descriptive and regression analyses were performed using the R Studio version 2023.12.0 Build 369.

### Data preprocessing for machine learning models

Binary variables such as female sex, NEC morphology, emergency presentation and late presentation were encoded using binary encoding (0 and 1), where a value of 1 indicated the presence of the feature, and 0 indicated its absence. Ordinal variables such as stage and IMD were encoded using ordinal encoding. Age, as a numerical variable, was scaled using standard scaling. Categorical variables such as race, geography, rurality and site were encoded using one-hot encoding.

### Development of XGBoost machine learning models

All eXtreme Gradient Boosting (XGBoost) models were developed using a random grid search for hyperparameter tuning and evaluated with five-fold cross-validation. The data were randomly split into 70% training and 30% testing sets for all XGboost models. Stratification by the outcome during splitting was only applied to binary XGBoost classifier models to maintain a proportional representation of the outcome variables in both sets. Area under the receiver operating characteristic curve (AUROC) was used to select the best-performing classifier XGBoost models, while the C-index (concordance index) was used to select the optimal survival-based XGBoost models from the hyperparameter search grid. A SHapley Additive exPlanations (SHAP) tree explainer beeswarm plot was used to explain all the machine learning models to identify the most important features. Cases with missing data were not excluded from the XGBoost models, as XGBoost can internally handle missing values (Stokanović *et al.* 2024). All codes for ML models were developed using the Python version 3.10.12. Libraries used included Pandas, NumPy and Scikit-learn.

### Survival analysis

The Kaplan–Meier (KM) estimator was used to generate KM plots. The log-rank test compared survival plots between each group. A univariable Cox regression analysis was performed and unadjusted hazard ratios (HRs) were generated with 95% confidence intervals. Statistically significant variables in the univariable analyses were included in multivariable Cox regression providing adjusted HR (aHR). A survival Cox-based machine learning XGBoost model was used to assess the factors associated with survival.

### Emergency versus routine presentation analysis

A univariable logistic regression analysis was conducted to identify factors associated with emergency presentation. A multivariable logistic regression analysis was then performed, including only the variables that showed statistical significance in the univariable logistic regression analyses. A binary classifier XGBoost model was developed to assess the factors associated with emergency presentation.

### Late versus early presentation analysis

A univariable logistic regression analysis was conducted to identify factors associated with late presentation. A multivariable logistic regression analysis was then performed, including only the variables that showed statistical significance in univariable logistic regression.

A binary classifier XGBoost model was developed to study the factors associated with late presentation.

### Development of a decision tree for factors affecting early and late presentations

A decision tree machine learning mode was developed to study the factors leading to late presentation of neuroendocrine neoplasms using demographic variables (age, sex, race, IMD, rurality and tumour site). Patients with missing data in these variables or the outcome variable (late presentation) were excluded. The dataset was split into training and testing sets (80:20 ratio) while stratifying the split using the outcome variable (late presentation). A random grid search of hyperparameters was performed to choose the most optimal decision tree model based on AUROC. Performance metrics were computed for training and testing datasets, including AUROC, accuracy, sensitivity, specificity, positive predictive value (PPV), negative predictive value (NPV) and F1 score. The model was developed using the RStudio version 2023.12.0 Build 369 and using rpart, rpart.plot, caret and pROC libraries. The decision tree was visualised to enhance clinical interpretability.

## Results

### Baseline characteristics

A total of 21,345 NEN cases were included (Table 1). Among them, 4,343 (20.3%) were diagnosed through the emergency route and 17,002 (79.6%) were diagnosed routinely. Most patients were aged over 55, with the most numerous age group 65–74 years (31.7%), followed by over 75 (24.8%). There was a near-equal sex distribution. Males tended to present as an emergency (55.1%) compared to females (44.9%). Stage 4 accounted for most of the cohort (28.2%), with emergency presentations representing the largest group diagnosed at stage 4 (37%). The majority were White (88.3%), compared to the population demographic of 81.7% White (Office for National Statistics 2022). The distribution was relatively uniform across regions and indices of IMD categories. Most patients were from predominantly urban areas (78.7%), with the highest frequency of cases in the South East (16.2%), followed by the North West (13.7%) and then London (12.2%) (Table 1). A map of England including population densities can be viewed on the Office of National Statistics website (Office for National Statistics 2021). In terms of tumour site, the most common site was lung (30.6%), followed by small intestine (26.8%) and then pancreas (18.5%). Statistically significant differences between the presentation groups were observed in all variables (Table 1).

**Table 1:** Baseline characteristics of the cohort.

		Overall	Emergency presentations	Routine presentations	*P*-value
		*n* (%)	*n* (%)	*n* (%)	
**Total**		**21,345 (100)**	**4,343 (20.3)**	**17,002 (79.7)**	
Sex					
	Male	10,612 (49.7)	2,393 (55.1)	8,219 (48.3)	**<0.001**
	Female	10,733 (50.3)	1,950 (44.9)	8,783 (51.7)	
Age					**<0.001**
	≤29	362 (1.7)	54 (1.2)	308 (1.8)	
	30–54	4,123 (19.3)	730 (16.8)	3,393 (20)	
	55–64	4,797 (22.5)	853 (19.6)	3,944 (23.2)	
	65–74	6,768 (31.7)	1,284 (29.6)	5,484 (32.3)	
	75+	5,295 (24.8)	1,422 (32.8)	3,873 (22.7)	
Ethnicity					**0.003**
	White	18,837 (88.3)	3,891 (89.6)	14,946 (87.9)	
	Asian	816 (3.8)	129 (3)	687 (4)	
	Black	529 (2.5)	93 (2.1)	436 (2.6)	
	Mixed	123 (0.6)	22 (0.5)	101 (0.6)	
	Other	303 (1.4)	69 (1.6)	234 (1.4)	
	Unknown	737 (3.5)	139 (3.2)	598 (3.5)	
IMD					**0.0006**
	5-least deprived	4,489 (21)	861 (19.8)	3,628 (21.3)	
	4	4,537 (21.3)	884 (20.4)	3,653 (21.5)	
	3	4,394 (20.6)	866 (19.9)	3,528 (20.8)	
	2	3,997 (18.7)	853 (19.6)	3,144 (18.5)	
	1-most deprived	3,928 (18.4)	879 (20.3)	3,049 (17.9)	
Region					**0.015**
	East Midlands	1,712 (8)	347 (8)	1,365 (8)	
	East of England	2,515 (11.8)	510 (11.7)	2,005 (11.8)	
	London	2,594 (12.2)	512 (11.8)	2,082 (12.2)	
	North East	1,299 (6.1)	272 (6.3)	1,027 (6)	
	North West	2,931 (13.7)	598 (13.8)	2,333 (13.7)	
	South East	3,452 (16.2)	639 (14.7)	2,813 (16.5)	
	South West	2,278 (10.7)	479 (11)	1,799 (10.6)	
	West Midlands	2,344 (11)	474 (10.9)	1,870 (11)	
	Yorkshire and the Humber	2,220 (10.4)	512 (11.8)	1,708 (10.2)	
Rurality					**0.052**
	Predominantly rural	4,269 (20)	822 (18.9)	3,447 (20.3)	
	Predominantly urban	16,792 (78.7)	3,453 (79.5)	13,339 (78.5)	
	Urban with significant rural	284 (1.3)	68 (1.6)	216 (1.2)	
Site					**<0.001**
	Caecum	734 (3.4)	202 (4.7)	532 (3.1)	
	Colon	784 (3.7)	224 (5.2)	560 (3.3)	
	Lung	6,534 (30.6)	939 (21.6)	5,595 (32.9)	
	Pancreas	3,955 (18.5)	707 (16.3)	3,248 (19.1)	
	Rectum	1,790 (8.4)	157 (3.6)	1,633 (9.6)	
	Small intestine	5,727 (26.8)	1,764 (40.6)	3,963 (23.3)	
	Stomach	1,821 (8.5)	350 (8)	1,471 (8.7)	
Morphology					**<0.001**
	NET	16,544 (77.5)	3,009 (69.3)	13,535 (79.6)	
	NEC	4,801 (22.5)	1,334 (30.7)	3,467 (20.4)	
Stage					**<0.001**
	1 (early presentation)	5,108 (23.9)	500 (11.5)	4,608 (27.1)	
	2 (early presentation)	1,667 (7.8)	335 (7.7)	1,332 (7.8)	
	3 (late presentation)	3,100 (14.5)	849 (19.5)	2,251 (13.2)	
	4 (late presentation)	6,009 (28.2)	1,608 (37)	4,401 (25.9)	
	Unknown	5,461 (25.6)	1,051 (24.3)	4,410 (26)	

### Survival analyses

**Figure 1:**
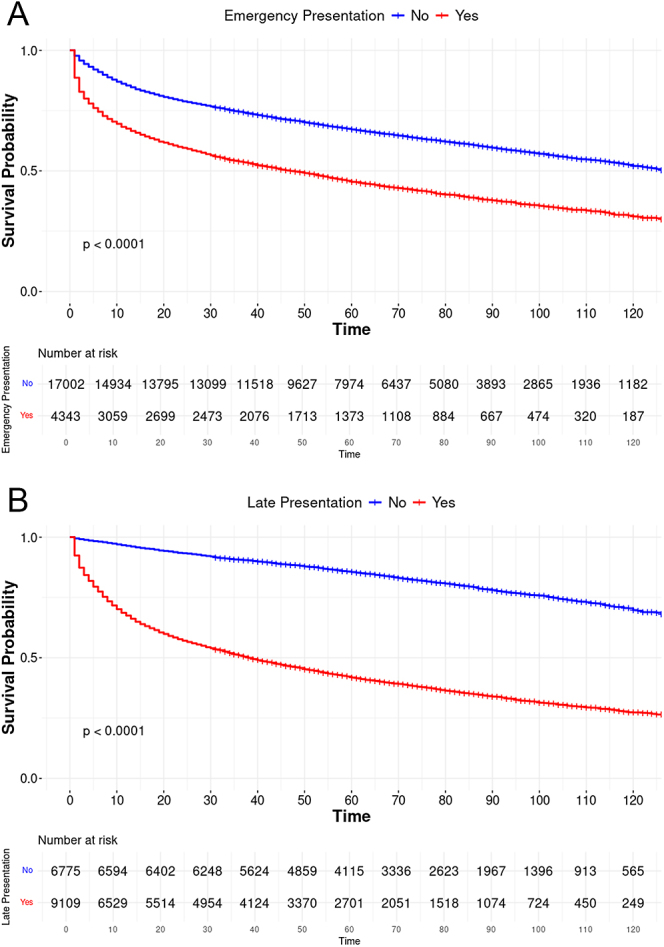
(A) Kaplan–Meier plot showing overall survival of emergency and routine presentation. (B) Kaplan–Meier plot showing overall survival of late and early presentation.

Figure 1A shows KM plots comparing the survival of patients presenting as emergencies or routinely showed a significant difference in survival curves between the groups on the log-rank test (*P* < 0.0001). Similarly, KM plots comparing late and early presentations showed a significant difference in survival (*P* < 0.0001) (Fig. 1B).

In the univariable Cox regression analyses, emergency presentation (HR 2.01, *P* ≤ 0.001), male sex, older age groups (HR 3.11–14.01, all *P* ≤ 0.001), NEC morphology (HR 4.70, *P* ≤ 0.001), advanced disease stage (HR 1.89–8.67, all *P* ≤ 0.001) and residing in more deprived areas (HR 1.09–1.22, all *P* ≤ 0.001) were significant predictors of worse survival. All other ethnicities showed lower hazards compared to White ethnicity. Residing in all other regions was associated with worse survival outcomes compared to London. All sites, excluding rectal NEN, had higher hazards compared to small intestinal NEN.

In the multivariable Cox regression analysis, emergency presentation (aHR 1.64, *P* ≤ 0.001), older age groups (aHR 2.12–7.51, all *P* ≤ 0.001), advanced stage (aHR 1.92–8.49, all *P* ≤ 0.001) and deprivation (aHR 1.09–1.25, *P* = 0.028 to *P* ≤ 0.001) remained significant predictors of worse OS. Female sex showed a reduced but still significant effect (aHR 0.82, *P* ≤ 0.001). The impact of ethnicity was less pronounced, with Black (aHR 0.80, *P* = 0.03) and Mixed (aHR 0.58, *P* = 0.014) ethnicities remaining significant with lower hazards compared to White counterparts. Geographical differences persisted, with all regions showing higher hazards compared to London. Compared to small intestinal NEN, all other NEN sites had statistically significantly worse survival (Table 2).

**Table 2:** Univariable and multivariable Cox regression analysis.

		Univariable Cox regression			Multivariable Cox regression		
		HR	95% CI	*P*-value	aHR	95% CI	*P*-value
Route	Routine	1 (ref)			1 (ref)		
	Emergency	2.01	1.92–2.11	**<0.001**	1.64	1.55–1.73	**<0.001**
Sex							
	Male	1 (ref)			1 (ref)		
	Female	0.70	0.68–0.73	**<0.001**	0.82	0.78–0.86	**<0.001**
Age							
	≤29	1 (ref)			1 (ref)		
	30–54	3.11	2.12–4.57	**<0.001**	2.12	1.41–3.18	**0.0003**
	55–64	5.31	3.63–7.77	**<0.001**	3.10	2.07–4.65	**<0.001**
	65–74	8.27	5.66–12.08	**<0.001**	4.55	3.04–6.82	**<0.001**
	75+	14.01	9.59–20.46	**<0.001**	7.51	5.02–11.23	**<0.001**
Ethnicity							
	White	1 (ref)			1 (ref)		
	Asian	0.58	0.51–0.66	**<0.001**	0.97	0.82–1.14	0.688
	Black	0.54	0.46–0.64	**<0.001**	0.80	0.66–0.98	**0.03**
	Mixed	0.55	0.39–0.78	**<0.001**	0.58	0.38–0.90	**0.014**
	Other	0.69	0.57–0.85	**<0.001**	0.86	0.68–1.08	0.184
	Unknown*						
IMD							
	5-least deprived	1 (ref)			1 (ref)		
	4	1.09	1.02–1.16	**0.006**	1.09	1.01–1.17	**0.028**
	3	1.13	1.06–1.21	**0.0001**	1.10	1.02–1.19	**0.013**
	2	1.14	1.07–1.22	**<0.001**	1.26	1.17–1.37	**<0.001**
	1-most deprived	1.22	1.15–1.31	**<0.001**	1.25	1.16–1.36	**<0.001**
Region							
	London	1 (ref)			1 (ref)		
	East Midlands	1.30	1.18–1.43	**<0.001**	1.21	1.07–1.36	**0.001**
	East of England	1.18	1.08–1.29	**0.0001**	1.15	1.03–1.27	**0.010**
	North East	1.25	1.12–1.39	**<0.001**	1.23	1.08–1.39	**0.001**
	North West	1.22	1.12–1.33	**<0.001**	1.15	1.03–1.27	**0.009**
	South East	1.23	1.13–1.33	**<0.001**	1.17	1.05–1.29	**0.002**
	South West	1.23	1.12–1.34	**<0.001**	1.14	1.02–1.27	**0.018**
	West Midlands	1.26	1.15–1.37	**<0.001**	1.16	1.05–1.29	**0.005**
	Yorkshire and the Humber	1.31	1.20–1.43	**<0.001**	1.21	1.09–1.36	**<0.001**
Rurality							
	Predominantly rural	1 (ref)					
	Predominantly urban	0.97	0.92–1.03	0.422			
	Urban with significant rural	1.07	0.89–1.28	0.440			
Site							
	Small intestine	1 (ref)			1 (ref)		
	Rectum	0.79	0.7251–0.8744	**<0.001**	2.82	2.51–3.17	**<0.001**
	Caecum	1.51	1.3611–1.6919	**<0.001**	1.57	1.39–1.77	**<0.001**
	Colon	2.46	2.2394–2.7124	**<0.001**	2.20	1.97–2.47	**<0.001**
	Lung	1.08	1.0271–1.1500	**0.003**	2.31	2.15–2.49	**<0.001**
	Pancreas	1.29	1.2174–1.3783	**<0.001**	1.88	1.74–2.04	**<0.001**
	Stomach	1.40	1.3028–1.5241	**<0.001**	2.42	2.18–2.70	**<0.001**
Morphology							
	NET	1 (ref)			1 (ref)		
	NEC	4.70	4.50–4.90	**<0.001**	2.89	2.73–3.05	**<0.001**
Stage							
	1	1 (ref)			1 (ref)		
	2	1.89	1.69–2.12	**<0.001**	1.92	1.71–2.16	**<0.001**
	3	2.74	2.51–2.99	**<0.001**	3.20	2.90–3.52	**<0.001**
	4	8.67	8.05–9.34	**<0.001**	8.49	7.81–9.24	**<0.001**
	Unknown*						

* Excluded from the Cox regression analysis.

**Figure 2:**
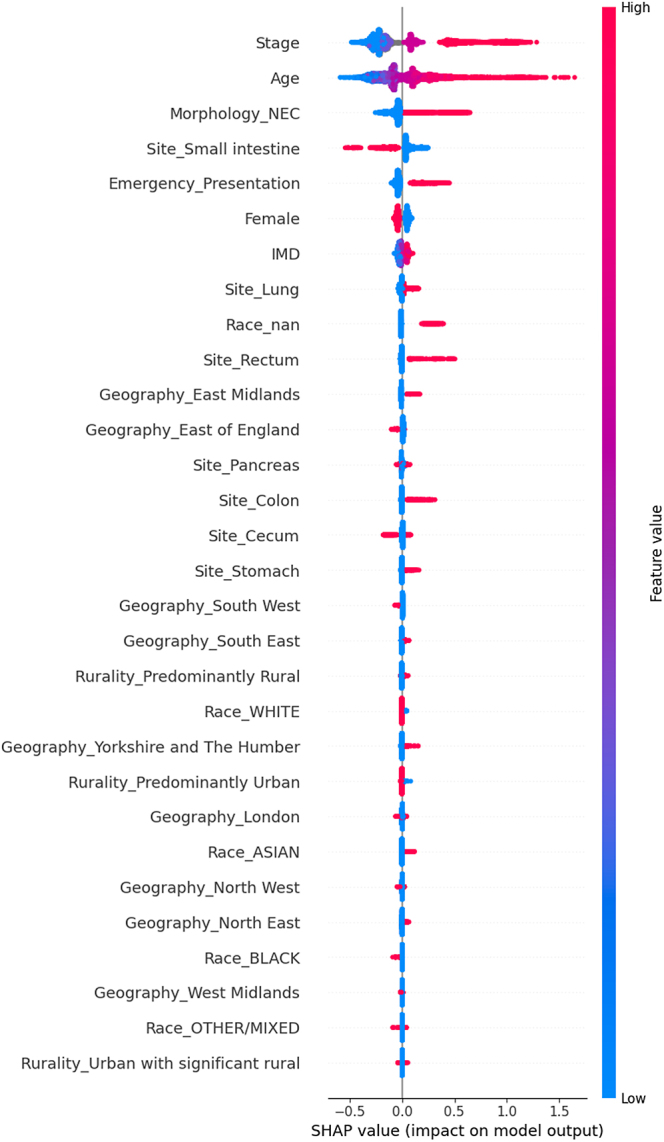
SHAP beeswarm plot for Cox-based survival XGBoost model to assess the factors associated with worse survival: this plot shows the top features influencing survival. Each dot represents a case, with red indicating higher feature values and blue indicating lower values. SHAP values (x-axis) quantify the impact on survival: points to the right of the midline indicate worse survival and points to the left of the midline indicate improved survival. For ordinal variables (e.g. stage), lower values are generally favourable. Binary variables (e.g. female sex and NEC morphology) reflect the impact of presence (1, red) versus absence (0, blue), and missing values are labelled as nan.

The SHapley Additive exPlanations for survival Cox-based XGBoost machine learning model showed that emergency presentation is the fifth most important factor in survival. The model demonstrated that, in order of decreasing importance, advanced stage, increasing age, NEC, emergency presentation, males, more deprived patients, lung NEN, rectal NEN, residing in East Midlands, colonic NEN, stomach NEN, and residing in Yorkshire and the Humber, and Asian race were associated with worse survival. Similarly, variables associated with better survival were early stage, younger age, NET, small intestinal NEN, routine presentations, female sex, less deprived patients, residing in East of England and the South West, and Black race in decreasing order of importance. The C-index for this model was 81.7% (training set) and 81.6% (testing set) (Fig. 2).

### Factors associated with emergency presentations

The multivariable logistic analysis revealed that females are less likely to be diagnosed as emergency presentation when compared to males (OR: 0.89, *P* = 0.005). Compared to stage 1, the odds of emergency presentation increased with advancing disease stage: stage 2 (OR: 1.80, *P* < 0.001), stage 3 (OR: 2.05, *P* < 0.001), and stage 4 (OR: 2.04, *P* < 0.001); however, stage 3 and 4 revealed similar association with emergency presentations. A significant increase in odds was observed in the most deprived group when compared to the least deprived (OR: 1.24, *P* < 0.001). Compared to London, a significant decrease in odds of emergency presentation was found in the North West (OR: 0.84, *P* = 0.038), South East (OR: 0.76, *P* = 0.001), and South West (OR: 0.82, *P* = 0.026). NEC was associated with a significantly higher likelihood of emergency presentations (OR: 1.71, *P* < 0.001) relative to NET. Compared to small intestinal NEN, caecal (OR = 0.75, *P* = 0.004), colonic (OR = 0.73, *P* = 0.002), lung (OR = 0.45, *P* < 0.001), pancreatic (OR = 0.53, *P* < 0.001), stomach (OR = 0.30, *P* < 0.001), and rectal NEN (OR = 0.60, *P* < 0.001) were associated with significantly lower odds of emergency presentation. There was no significant association between emergency presentation and rurality in the univariable analysis, and no significant association between emergency presentation and age (except in aged 65–74) or ethnicity in the multivariable analysis (Table 3).

**Table 3:** Univariable and multivariable logistic regressions for factors affecting emergency and late presentations.

		Outcome: emergency presentation						Outcome: late presentation					
		Univariable logistic regression			Multivariable logistic regression			Univariable logistic regression			Multivariable logistic regression		
		OR	95% CI	*P*-value	aOR	95% CI	*P*-value	OR	95% CI	*P*-value	aOR	95% CI	*P*-value
Sex													
	Male	1 (ref)			1 (ref)			1 (ref)			1 (ref)		
	Female	0.76	0.71–0.82	**<0.001**	0.89	0.82–0.97	**0.005**	0.59	0.55 0.63	**<0.001**	0.78	0.72–0.85	**<0.001**
Age													
	≤29	1 (ref)			1 (ref)			1 (ref)			1 (ref)		
	30–54	1.23	0.92–1.67	0.181	0.78	0.55–1.13	0.18	3.29	2.44 4.50	**<0.001**	2.34	1.64–3.41	**<0.001**
	55–64	1.23	0.92–1.68	0.168	0.71	0.50–1.03	0.065	4.63	3.44 6.32	**<0.001**	3.01	2.11–4.37	**<0.001**
	65–74	1.34	1.00–1.81	0.168	0.69	0.49–1.00	**0.04**	5.37	4.00 7.32	**<0.001**	3.23	2.26–4.67	**<0.001**
	75+	2.09	1.57–2.84	**<0.001**	0.99	0.70–1.43	0.962	6.31	4.69 8.63	**<0.001**	3.49	2.44–5.07	**<0.001**
Ethnicity													
	White	1 (ref)			1 (ref)			1 (ref)			1 (ref)		
	Asian	0.72	0.59–0.87	**0.0008**	0.9	0.69–1.17	0.441	0.49	0.41 0.60	**<0.001**	0.55	0.43–0.70	**<0.001**
	Black	0.82	0.65–1.02	0.084	0.78	0.57–1.05	0.104	0.64	0.52 0.79	**<0.001**	0.87	0.67–1.15	0.331
	Mixed	0.84	0.51–1.30	0.449	0.94	0.50–1.65	0.832	0.75	0.48 1.18	0.214	1.03	0.58–1.80	0.926
	Other	1.13	0.86–1.48	0.367	1.3	0.93–1.80	0.114	0.9	0.69 1.18	0.446	1.29	0.93–1.80	0.13
	Unknown*												
IMD													
	5-least deprived	1 (ref)			1 (ref)			1 (ref)					
	4	1.02	0.92–1.13	0.714	0.99	0.87–1.12	0.898	1	0.90 1.10	0.934			
	3	1.03	0.93–1.15	0.529	1.01	0.89–1.14	0.938	1	0.91 1.10	0.988			
	2	1.14	1.03–1.27	**0.013**	1.11	0.97–1.26	0.125	1.03	0.93 1.13	0.614			
	1-most deprived	1.21	1.09–1.35	**<0.001**	1.24	1.09–1.42	**0.001**	1.04	0.94 1.15	0.614			
Region													
	London	1 (ref)			1 (ref)			1 (ref)			1 (ref)		
	East Midlands	1.03	0.89–1.20	0.669	0.87	0.72–1.06	0.171	1.26	1.09 1.47	**0.002**	1.05	0.86–1.27	0.650
	East of England	1.03	0.90–1.19	0.629	0.94	0.79–1.11	0.451	1.05	0.92 1.19	0.452	0.87	0.74–1.03	0.112
	North East	1.08	0.91–1.27	0.378	0.85	0.69–1.05	0.13	1.14	0.97 1.34	0.106	0.93	0.76–1.15	0.517
	North West	1.04	0.91–1.19	0.538	0.84	0.71–0.99	**0.038**	1.21	1.07 1.38	**0.003**	1.01	0.85–1.19	0.952
	South East	0.92	0.81–1.05	0.229	0.76	0.64–0.90	**0.001**	1.15	1.02 1.30	**0.024**	0.94	0.80–1.11	0.484
	South West	1.08	0.94–1.24	0.264	0.82	0.68–0.98	**0.026**	1.41	1.23 1.61	**<0.001**	1.09	0.91–1.30	0.348
	West Midlands	1.03	0.90–1.19	0.67	0.85	0.71–1.01	0.068	1.3	1.14 1.49	**0.0001**	0.95	0.80–1.13	0.586
	Yorkshire and the Humber	1.22	1.06–1.40	**0.004**	0.91	0.76 1.09	0.303	1.22	1.06 1.40	**0.004**	0.94	0.78–1.12	0.490
Rurality													
	Predominantly rural	1 (ref)						1 (ref)					
	Predominantly urban	1.09	1.00–1.18	0.057				0.98	0.91 1.06	0.59			
	Urban with significant rural	1.32	0.99–1.74	0.054				1.14	0.86 1.51	0.365			
Site													
	Small intestine	1 (ref)			1 (ref)			1 (ref)			1 (ref)		
	Caecum	0.85	0.72–1.01	0.069	0.75	0.62–0.91	**0.004**	2	1.50–2.74	**<0.001**	1.69	1.25–2.33	**<0.001**
	Colon	0.9	0.76–1.06	0.203	0.73	0.59–0.89	**0.002**	0.84	0.67–1.07	**<0.001**	0.38	0.29–0.49	**<0.001**
	Lung	0.38	0.34–0.41	**<0.001**	0.45	0.40–0.51	**<0.001**	0.09	0.08–0.10	0.146	0.05	0.05–0.06	**<0.001**
	Pancreas	0.49	0.44–0.54	**<0.001**	0.53	0.46–0.60	**<0.001**	0.24	0.21–0.27	**<0.001**	0.19	0.17–0.21	**<0.001**
	Stomach	0.53	0.18–0.26	**<0.001**	0.30	0.24–0.38	**<0.001**	0.1	0.09–0.12	**<0.001**	0.05	0.05–0.06	**<0.001**
	Rectum	0.22	0.47–0.61	**<0.001**	0.60	0.50–0.73	**<0.001**	0.23	0.20–0.27	**<0.001**	0.1	0.08–0.12	**<0.001**
Morphology													
	NET	1 (ref)			1 (ref)			1 (ref)			1 (ref)		
	NEC	1.73	1.61–1.86	**<0.001**	1.71	1.54–1.90	**<0.001**	5.1	4.66 5.6	**<0.001**	10.18	9.16–11.34	**<0.001**
Stage													
	1	1 (ref)			1 (ref)								
	2	2.32	1.99–2.70	**<0.001**	1.8	1.53–2.12	**<0.001**						
	3	3.48	3.08–3.93	**<0.001**	2.05	1.78–2.37	**<0.001**						
	4	3.37	3.02–3.76	**<0.001**	2.04	1.79–2.33	**<0.001**						
	Unknown*												
Route	Routine							1 (ref)					
	Emergency							2.63	2.41 2.86	**<0.001**	1.54	1.38–1.71	**<0.001**

* Excluded from the logistic regression analysis.

**Figure 3:**
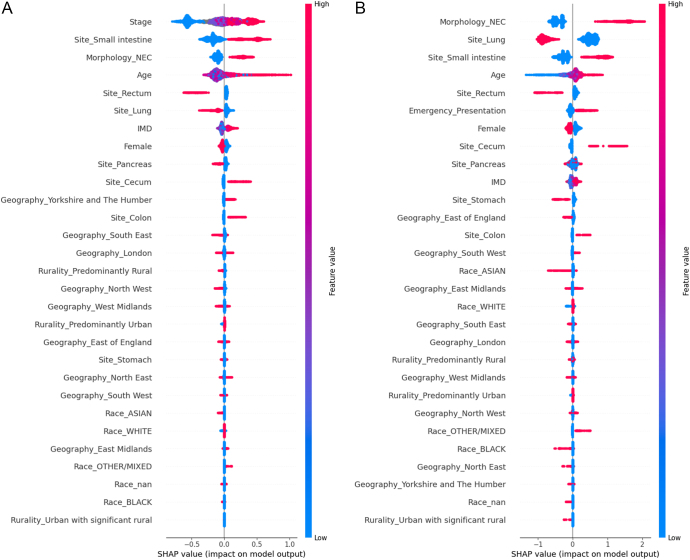
(A) SHAP beeswarm plot for binary XGBoost classifier model for assessing factors associated with routine and emergency presentation: this plot shows the top features influencing routine and emergency presentations. Each dot represents a case, with red indicating higher feature values and blue indicating lower values. SHAP values (x-axis) quantify the impact on emergency presentation: points to the right of the midline indicate emergency presentation and points to the left of the midline indicate routine presentation. For ordinal variables (e.g. stage), lower values are generally favourable. Binary variables (e.g. female sex and NEC morphology) reflect the impact of presence (1, red) versus absence (0, blue), and missing values are labelled as nan. (B) SHAP beeswarm plot for binary XGBoost classifier model for assessing factors associated with early and late presentation: this plot shows the top features influencing early and late presentation. Each dot represents a case, with red indicating higher feature values and blue indicating lower values. SHAP values (x-axis) quantify the impact on emergency presentation: points to the right of the midline indicate late presentation and points to the left of the midline indicate early presentation. For ordinal variables (e.g. stage), lower values are generally favourable. Binary variables (e.g. female sex and NEC morphology) reflect the impact of presence (1, red) versus absence (0, blue), and missing values are labelled as nan.

The SHapley Additive exPlanations for binary XGBoost classifier model revealed that emergency presentation is associated with advanced stage, small intestinal NEN, NEC, advanced age, deprivation, caecal NEN, residing in Yorkshire and the Humber, colonic NEN, and mixed race in decreasing order of importance. The model also showed that early-stage, NET, rectal NEN, lung NEN, female sex, pancreatic NEN, residing in South East England, rural dwellers, residing in North West England, and Asian race were associated with routine presentation, in decreasing order of importance. The accuracy of this model was 80% (testing set) and 80% (training set). The AUROC was 68.2% (testing set) and 73.8% (training set) (Fig. 3A).

### Factors associated with late presentation

The multivariable logistic analysis revealed that emergency presentations were more likely to be late stage (stage 3 or 4) (OR: 1.54, *P* < 0.001). Females were less likely to present late compared to males (OR: 0.78, *P* < 0.001). Compared to individuals aged 29 years or younger, those in the 30–54 (OR: 2.34, *P* < 0.001), 55–64 (OR: 3.01, *P* < 0.001) and 65–74 (OR: 3.23, *P* < 0.001) years age groups were progressively more likely to present with advanced stage. Asians were significantly less likely to present late (OR: 0.55, *P* < 0.001). NEC was associated with a higher likelihood of late presentation (OR: 10.18, *P* < 0.001) relative to NET. Compared to small intestinal NEN, caecal NEN was associated with significantly higher odds of late presentation (OR = 1.69, 95% CI: 1.25–2.33, *P* < 0.001). In contrast, colonic (OR = 0.38, *P* < 0.001), lung (OR = 0.05, *P* < 0.001), pancreatic (OR = 0.19, *P* < 0.001), stomach (OR = 0.05, *P* < 0.001) and rectal NEN (OR = 0.10, *P* < 0.001) were associated with significantly lower odds of late presentation. There was no significant association between late presentation and IMD or rurality in univariable analysis. There was no significant association between late presentation and geographical region in multivariable analysis (Table 3).

The SHapley Additive exPlanations for binary XGBOOST classifier model revealed that late presentations are associated with NEC morphology, small intestinal NEN, advanced age, emergency presentation, caecal NEN, colon NEN and mixed race in decreasing order of importance. It also revealed that NET morphology, lung NEN, younger age, rectal NEN, stomach NEN, East of England, and Asian and Black race are associated with early presentations in decreasing order of importance. The accuracy of this model was 79% (testing set) and 80% (training set). The AUROC was 85.1% (testing set) and 86.5% (training set) (Fig. 3B). A decision tree machine learning model was developed to predict late and early presentation of NENs. This model only included pre-diagnostic variables (demographic), hence excluded morphology (Supplementary Figs 1 and 2 (see section on Supplementary materials given at the end of the article)). Metrics for the decision tree machine learning model were AUROC (77.4%, 77.3%), accuracy (71.6%, 71.3%), sensitivity (64.1%, 65.5%), specificity (77.1%, 75.6%), PPV (67.5%, 66.5%), NPV (74.3%, 74.7%) and F1 score (65.8%, 66.0%) for training and testing sets, respectively. A summary of the findings from the regressions and ML models is presented in Supplementary Table 1.

## Discussion

### Summary of main findings

The findings suggest that emergency presentations are associated with NEC morphology and advanced stage. Males tend to be diagnosed following emergency presentations more than females. As expected, advanced stage was more associated with older age. Patients living in the most deprived areas are more likely to be diagnosed after emergency presentations when compared with the least deprived. Routine presentations were more common in the North West, South East and South West of England compared to London. Residents of London had better survival compared to other regions.

Small intestinal and caecal NEN present with advanced disease. Small intestinal NEN had the most favourable survival but paradoxically present more often as emergencies, possibly due to small intestinal bowel lumen being more susceptible to obstructive symptoms on clinical presentation than, for example, NEN of the caecum. Lung, stomach and rectal NENs were more likely to present with early-stage disease. Rectal NEN had unfavourable survival yet appeared to present routinely, suggesting NEC may be influencing this site, as has been shown previously (White *et al.* 2022). Lung and stomach NEN had worse survival (compared to small intestinal NEN). However, lung NENs tend to present routinely, and this may reflect the increased frequency of CT scanning of the chest for mild symptoms.

### Implications

The findings suggest that prioritising access to healthcare and screening, particularly for older age groups, males may lead to more timely diagnoses and improved survival outcomes. Improved screening strategies are needed for small intestinal and caecal NEN as they present late and as emergencies. However, compared to small intestinal NEN, caecal NEN was associated with significantly higher odds of late presentation.

Despite being detected early and routinely, rectal NENs have unfavourable survival outcomes, suggesting the need for further pathological and/or epidemiological studies to investigate this paradoxical trend. Other potential interventions may include educational programmes targeting primary care health practitioners to improve recognition of NEN presentations for earlier diagnoses. The findings suggest a degree of variation between geographical areas, which together with other factors identified should be relayed to health commissioners to set appropriate mitigating measures and strategies.

### Demographic targets for interventions

Although no significant differences in incidence rates between males and females are observed in the literature, a significant survival advantage is observed for females in NEN, which aligns with the current findings (Caldarella *et al.* 2011, Boyar Cetinkaya *et al.* 2018, White *et al.* 2023, Zheng *et al.* 2023, Beck *et al.* 2024, Mortagy *et al.* 2024). The reasons for the survival disadvantage among males remain unclear. However, the literature highlights behavioural factors and cultural norms that lead males to seek medical care less frequently and to be reluctant to discuss health concerns, contributing to delays in diagnosis, as evidenced by the current findings (Schlichthorst *et al.* 2016, Ruggeri *et al.* 2022). Behavioural differences alone are unlikely to be the sole contributors to the observed differences in survival, as other factors related to hormonal receptor expression have also been studied (Sica *et al.* 2008, Estrella *et al.* 2014). Targetable are behavioural aspects; thus, public health campaigns encouraging males to undergo health screening may be beneficial. As expected, older age groups are associated with progressively worse survival outcomes in NEN (White *et al.* 2023). Screening programmes (e.g. CT scanning) may result in a higher pickup of NEN. However, it is known that screening programmes have a higher rate of uptake in less deprived populations, suggesting that a greater focus should be put on health campaigns aimed at increasing uptake in more deprived areas, which may be better achieved using community outreach services such as mobile screening units.

Revisiting current screening guidelines and potentially lowering the age cut-offs for eligibility may improve early detection while considering cost-effectiveness. Training healthcare providers on symptom recognition of NEN at the primary care level is crucial. It is important to ensure that symptoms in older age groups are not overlooked and are addressed appropriately. For instance, flushing episodes in older females are often attributed to menopause (Ravikumar *et al.* 2021). Deprived communities were linked to increased EP and worse outcomes in NEN in the findings. Implementing screening programs through mobile units or community awareness outreach campaigns could help educate individuals about the symptoms of NEN and improve access to diagnostic tests, which would include gastroscopy/colonoscopy and CT scans. Slight geographical disparities were observed, hence, encouraging local authorities to share best practices regarding health screening, access and awareness may help reduce these variations.

### Tumour sites and screening strategies

Small intestinal and caecal NEN appeared to be linked to advanced stage at diagnosis and should be targeted for earlier detection. NENs are thought to be often misdiagnosed as their symptoms mimic other gastrointestinal disorders, and common diagnostic tools such as CT or MRI scans are usually utilised after significant delays when symptoms persist (Pavel *et al.* 2015, Singh *et al.* 2017). There is minimal evidence on the duration of presenting symptoms of patients with NENs leading to delayed diagnoses (Feinberg *et al.* 2013); however, a study that explored symptoms and access to healthcare before diagnosis by surveying patients (most of which were diagnosed with small intestinal NEN) estimated time from first symptom to diagnosis was 53 months, with most of the respondents rating their initial symptoms as severe (Basuroy *et al.* 2018). Despite being diagnosed later, small intestinal NEN had the best survival, which may point towards the analysis of small intestinal NEN and how they differ from advanced NEN at other sites.

In England, bowel screening using the faecal immunochemical test begins at the age of 54 and continues up to 74 years, whereas in the US, screening typically starts at the age of 50 and continues until 75 years (NHS 2024, U.S. Preventive Services Task Force 2021). Comparison of survival rates for NENs showed better overall survival for caecal, colon and rectal NENs in the US compared to England, potentially due to differences in screening strategies (Mortagy *et al.* 2024). The program, however, is expanding to include ages 50–74 years, which could help detect cases earlier (NHS 2024).

Tumour sites that appeared to present routinely and with earlier disease stage disease were lung and rectal NEN. Lung cancer screening strategies in the US and England are similar, and previous literature shows comparable survival rates for lung NEN patients (Centers for Disease Control and Prevention 2024, Page 2024, Mortagy *et al.* 2024). Despite presenting routinely, lung and rectal NENs have poor survival, which is a paradoxical finding. This implies that NEN of the rectum or lung may be targeted for basic science research. These sites of NEN are the same sites found to have significantly worse survival for males (White *et al.* 2023). This suggests there may be further benefit to investigating differences in tumour microenvironment influenced by sex hormones, in addition to behavioural and cultural factors. Unlike other solid organ cancers where one set of risk factors can be identified, the NEN of each organ site likely has its own unique set of risk factors. Hence, examining the differences in survival based on the factors studied lends weight to the theory that the NEN of each different solid organ site should be studied in greater detail. Rectal and lung NENs are clinically known to present in two different groups – one group consists of relatively benign grade 1 and small tumours, and the other consists of higher grade tumours that behave in a more malignant way. These tumours present as two groups (early and late stages) that behave differently, which was observed in the decision tree machine learning model. Depending on age, sex and IMD status, lung and rectal NEN may present either early or late (see Supplementary Figs 1 and 2).

### Strengths and limitations

To the authors’ knowledge, no previous study has assessed the differences in survival between NEN patients presenting as emergency cases and those presenting routinely, nor the underlying factors affecting emergency and late presentations. This study also includes the largest number of NEN patients collected robustly. Limitations included the absence of tumour size and grade, which were excluded due to a high percentage of missing data, the lack of Ki67 data, quality of life metrics, and the unavailability of information on patient symptoms and co-morbidities in primary care, as these are not reported in NCRAS. Accurate reporting of grade tumours and Ki67 will improve reporting on grading classification and prognosis in future analyses. Most of the patients included in this analysis were of White ethnicity, which may not be generalisable to other populations. In addition, it is difficult to conclude differences between racial groups due to the limited representation of non-White ethnicities in the dataset. The geographical areas used in the analysis were limited to England and were broad, which may not capture variations between smaller regions and other parts of the UK. The analysis used machine learning models alongside other statistical methods to identify subgroups and characteristics needing targeted interventions for improved outcomes, serving as a validation where the findings aligned. The findings of this study may not be generalisable to healthcare systems with different primary care and screening strategies. Machine learning models developed in this study are not externally validated, which limits their generalisability to other datasets and populations. As a result, the models should be considered exploratory tools rather than definitive predictive models. In addition, some outcomes in the study, such as emergency presentation, were imbalanced, which could lead to biased models favouring the majority class. This may affect the reliability of performance metrics, such as AUROC, and limit the model’s ability to accurately predict minority class outcomes. A limitation of SHapley Additive exPlanations (SHAP) is that while it provides valuable insights into feature importance, it does not establish causal relationships. Some differences were found between the outputs of the classical models and ML models, likely due to data heterogeneity. This was addressed by focussing on the validated findings of both models. Future work should aim to validate these results using independent or prospective datasets to confirm their generalisability.

## Conclusion

Emergency presentations and advanced disease stage at diagnosis are associated with poor survival. Health awareness and participation in screening programmes for at-risk groups should ideally be prioritised to improve timely diagnosis, decrease emergency presentation and improve survival in NEN.

## Supplementary materials



## Declaration of interest

The authors declare that there is no conflict of interest that could be perceived as prejudicing the impartiality of the work reported.

## Funding

This study was not funded. MLEA is a fellow partly funded by the Wessex Comprehensive Research Network.

## Author contribution statement

All authors provided substantial contributions to the conception design, acquisition and interpretation of study data and approved the final version of the paper.

## Data availability

NCRAS: DARS-NIC-656877-H3Z0P; the data is only available on applying through the Data Access Request Service (DARS) (https://digital.nhs.uk/services/data-access-request-service-dars) which is administered by NHS England.

## Ethics approval statement

Ethics clearance and informed patient consent were not required as the study involved secondary data analysis of anonymised patient data from NCRAS (England). The overall project is covered by ethics clearance from the National Health Service Research Ethics Committee Integrated Research Application System (NHS REC-IRAS number: 284875).
