# Supplementary material for: Emergency and routine presentation of neuroendocrine neoplasia in England: determinants of late presentation and survival outcomes
Source: Endocr Oncol. 2025 Apr 14;5(1):e250012. doi: 10.1530/EO-25-0012 (PMC12020462; doi:10.1530/EO-25-0012)
Supplement: Supplementary file 1 [file supplementary_materials.pdf]

Supplementary Table 1: Summary of findings from the multivariable regression and machine learning (ML) models*

Variable	Survival Cox	Survival ML	Early vs. Late logistic	Early vs. Late logistic ML	Emergency vs. Routine logistic	Emergency vs. Routine ML
**Male**	Worse (compared to females)	Worse	Late (compared to females)	Late	Emergency (compared to females)	Emergency
**Advanced age**	Worse	Worse	Late	Late	NS	Emergency
**Asian**	Similar to white	Worse	Early (compared to white)	Early	NS (compared to white)	Routine
**Black**	Improved relative to White	Better	NS (compared to white)	Early	NS (compared to white)	NS
**Mixed**	Improved relative to White	NS	NS (compared to white)	Late	NS (compared to white)	Emergency
**Most deprived**	Worse	Worse	NS (compared to white)	NS	Emergency	Emergency
**Small intestine (SI)**	Most favorable	Better	Late	Late	Emergency	Emergency
**Rectum**	Reduced relative to Small Intestine	Worse	Early (compared to SI)	Early	Routine	Routine
**Cecum**	Reduced relative to Small Intestine	NS	Late (compared to SI)	Late	Routine	Emergency
**Colon**	Reduced relative to Small Intestine	Worse	Early (compared to SI)	Late	Routine	Emergency
**Lung**	Reduced relative to Small Intestine	Worse	Early (compared to SI)	Early	Routine	Routine
**Pancreas**	Reduced relative to Small Intestine	NS	Early (compared to SI)	NS	Routine	Routine
**Stomach**	Reduced relative to Small Intestine	Worse	Early (compared to SI)	Early	Routine	NS
**NEC**	Worse (compared to NET)	Worse	Late (compared to NET)	Late	Emergency	Emergency
**Advanced stage**	Worse	Worse	---	---	Emergency	Emergency
**London**	Most favourable	NS	NS	NS	NS	NS
**East Midlands**	Reduced relative to London	Worse	NS (similar to London)	NS	NS (compared to London)	NS

**East of England**	Reduced relative to London	Better	NS (similar to London)	Early	NS (compared to London)	NS
**North East**	Reduced relative to London	Worse	NS (similar to London)	Early	NS (compared to London)	NS
**North West**	Reduced relative to London	NS	NS (similar to London)	NS	Routine (compared to London)	Routine
**South East**	Reduced relative to London	Worse	NS (similar to London)	NS	Routine (compared to London)	Routine
**South West**	Reduced relative to London	Better	NS (similar to London)	Late	Routine (compared to London)	NS
**West Midlands**	Reduced relative to London	NS	NS (similar to London)	NS	NS (compared to London)	NS
**Yorkshire and The Humber**	Reduced relative to London	Worse	NS (similar to London)	NS	NS (compared to London)	Emergency

NS: Non-significant association, ML: Machine learning model

***The use of classical and machine learning models**

This study used both classical models (Cox and logistic regression models) and machine learning models (binary and survival XGBoost) to analyse determinants of outcomes. This dual approach aimed to validate the findings from the classical models using the strengths of machine learning models. Classical models offer transparent, interpretable results traditionally used in clinical research. ML models complement these models by capturing complex and non-linear interactions and ranking variables by importance.

Both models largely agreed on key determinants. However, there were some differences between classical models and ML models especially in ethnicity, geographical variables and some anatomical site variables (Supplementary Table 1). Differences in ethnicity and geography could be due to varying access to healthcare or treatment options, environmental and lifestyle factors, or data heterogeneity. These differences do not undermine the study's main findings but highlight the potential of ML to detect the nuances beyond the scope of classical models and may inform future research. To ensure robustness, this study focused on the findings validated in both approaches while acknowledging the complementary insights provided by ML. These findings offered actionable insights into factors associated with patient outcomes which could help guide healthcare resource allocation.

Supplementary Figure 1: Visualisation of a decision tree machine learning model for the demographic factors leading to early and late presentation of neuroendocrine neoplasms

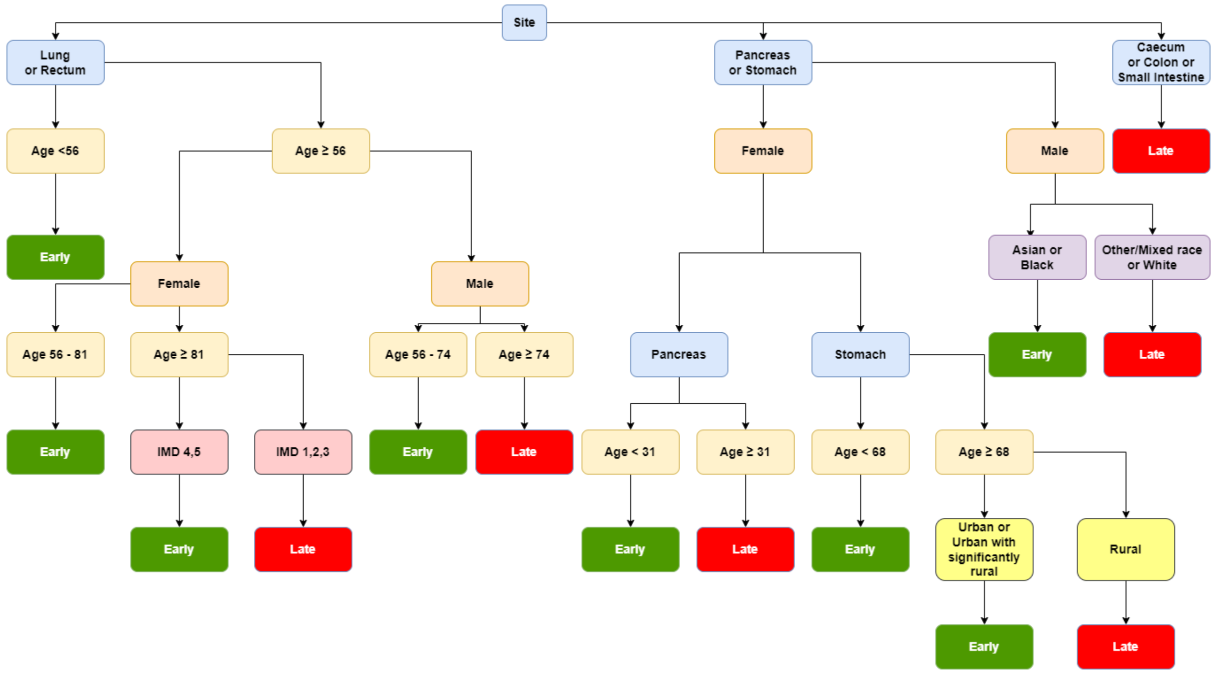

Supplementary Figure 2: Graphical summary of the decision tree represented in

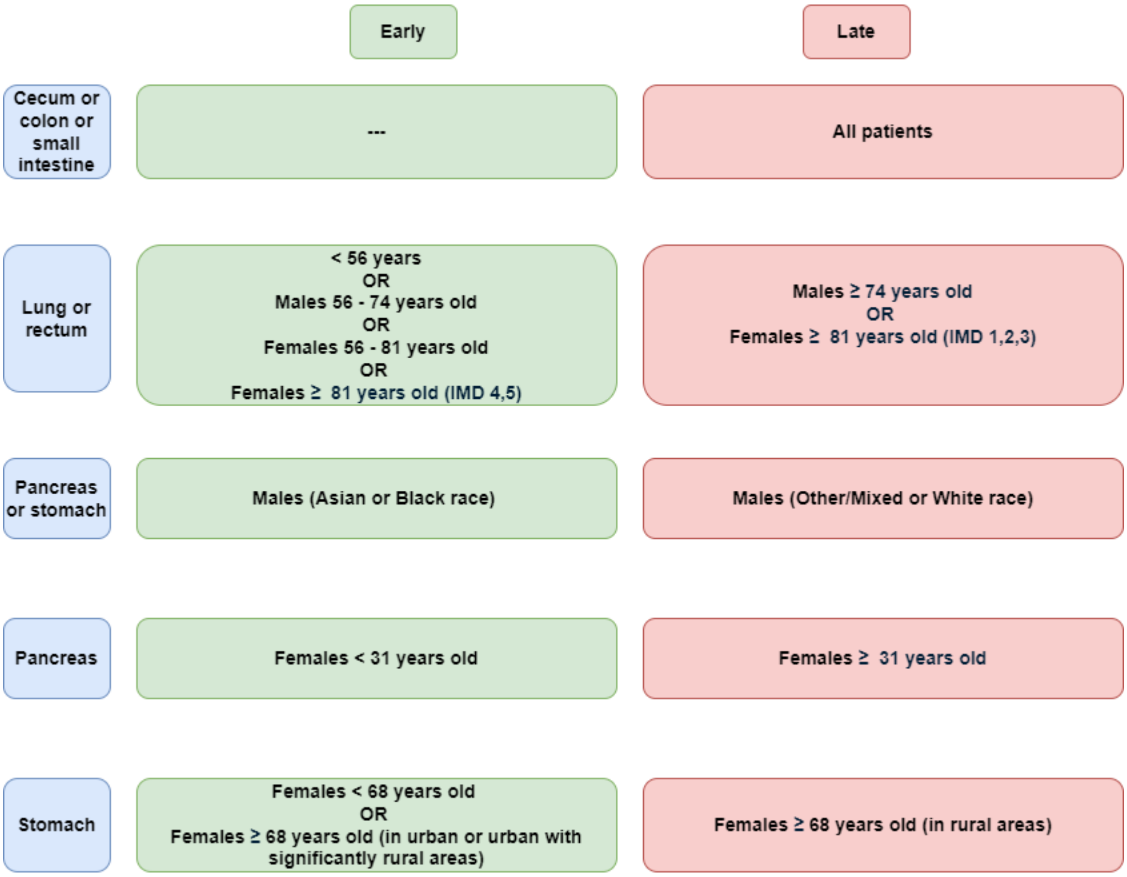

Supplementary Figure 1
